# Correlation between the Immune Checkpoint Inhibitors Prognostic Index and Outcomes in Nonsmall Cell Lung Cancer: A Multicentre Analysis

**DOI:** 10.1155/2022/7050817

**Published:** 2022-08-26

**Authors:** Ying Zhou, Bin Wu, Tian Li, Yong Zhang, Tianqi Xu, Ning Chang, Jian Zhang

**Affiliations:** ^1^Department of Respiratory and Critical Care Medicine, Xijing Hospital, Fourth Military Medical University, Xi'an 710032, Shannxi, China; ^2^School of Basic Medicine, Fourth Military Medical University, Xi'an 710032, Shannxi, China

## Abstract

**Objective:**

To evaluate the prognostic value of the immune checkpoint inhibitor prognostic index (ICPI), based on the albumin (ALB) and derived neutrophil-to-lymphocyte ratio (dNLR), for nonsmall cell lung cancer (NSCLC) patients receiving immune checkpoint inhibitors (ICIs).

**Methods:**

We conducted a multicentre retrospective study with an ICIs cohort (*n* = 143) and a chemotherapy control cohort (*n* = 84). A Cox proportional hazards regression and logistic regression model were used to find the independent risk factor for progression-free survival (PFS) and overall survival (OS) and disease control rate (DCR) in NSCLC patients. The Kaplan–Meier was used to evaluating the PFS and OS.

**Results:**

The ALB <35 g/L and dNLR >3 were correlated with worse PFS and OS for NSCLC patients receiving ICIs, respectively. The moderately high-risk ICPI had a significantly increased risk of progression (hazard ratio (HR) 1.83, 95% confidence interval (CI) 1.14–2.91; *P*=0.012) and of death (HR 2.33, 95% CI 1.12–4.87; *P*=0.024) and of nondisease control (odds ratio (OR) 3.05, 95% CI 1.19–7.83; *P*=0.021) and was correlated with worse PFS and 1-year survival rates (4.0 months vs. 7.2 months; *P*=0.001; 44.3% vs. 76.1%; *P*=0.001) compared with low-risk ICPI when it was characterized two groups. When ICPI was further divided into three groups, the results showed that the high-risk ICPI was correlated with worse PFS and 1-year survival rates. However, there was no difference in the chemotherapy cohort.

**Conclusion:**

The ICPI was correlated with worse outcomes for NSCLC patients receiving ICIs but not for patients with chemotherapy.

## 1. Introduction

The success of immunotherapy has not only revolutionized the pattern but also the landscape of nonsmall cell lung cancer (NSCLC) treatment [1]. The immune checkpoint inhibitors (ICIs), principally represented by cytotoxic T lymphocyte antigen-4 and programmed death 1/ligand 1 (PD-1/PD-L1) inhibitors, have been widely and successfully used in clinical practice [2]. Increasing evidence shows that up to 80% of NSCLC patients do not benefit from ICIs [3], and what is more, some patients even develop severe immunotoxicity and financial toxicity, although biomarkers promise new dawn for patients.

The tumor-related biomarkers, such as PD-L1 expression are widely used in clinical applications. A correlation between high PD-L1 expression and good outcomes has been observed in NSCLC patients receiving ICIs. In contrast, some studies showed that nearly 60–70% of patients did not benefit from ICIs even in the PD-L1 positive population [4–6]. In some circumstances, some patients show clinical benefits regardless of the expression level of PD-L1 in tumor cells [7]. Besides, PD-L1 has no uniform detection platform and cutoff value [5, 8]. Another biomarker is tumor mutation burden (TMB). Numerous studies indicate that patients with high TMB have a higher overall response rate (ORR), progression-free survival (PFS), and overall survival (OS) [9, 10]. However, the limitations of TMB are salient, including costly and time-consuming detection, and a lack of a standardized detection platform and uniform cutoff value. The imperfections of these tumor-related biomarkers are becoming increasingly apparent.

An increasing amount of research has confirmed that the systemic inflammatory response (SIR) is inextricably related to the occurrence and development of tumors, and also affects the immune response of cancer, which may be associated with the effect of immunotherapy [11–13]. Numerous routine blood parameters have been demonstrated as SIR-related biomarkers such as circulating white blood cells (WBC), absolute neutrophil counts (ANC), platelet counts (PLT), lactate dehydrogenase (LDH), albumin (ALB), and even neutrophil-to-lymphocyte ratio (NLR) [14], which were associated with poor prognosis in several malignant solid tumors, including NSCLC [15]. However, the prognostic and predictive value of SIR-related biomarkers in NSCLC with ICIs has not yet been completely elucidated. In the present study, we sought to explore a novel, convenient, practical, and economical combined prognostic index to predict the outcomes of NSCLC patients receiving ICIs, and help clinicians determine and screen NSCLC patients who are ineligible for ICIs in order to avoid unnecessary immunotoxicity and financial toxicity.

## 2. Materials and Methods

### 2.1. Study Population

**Figure 1:**
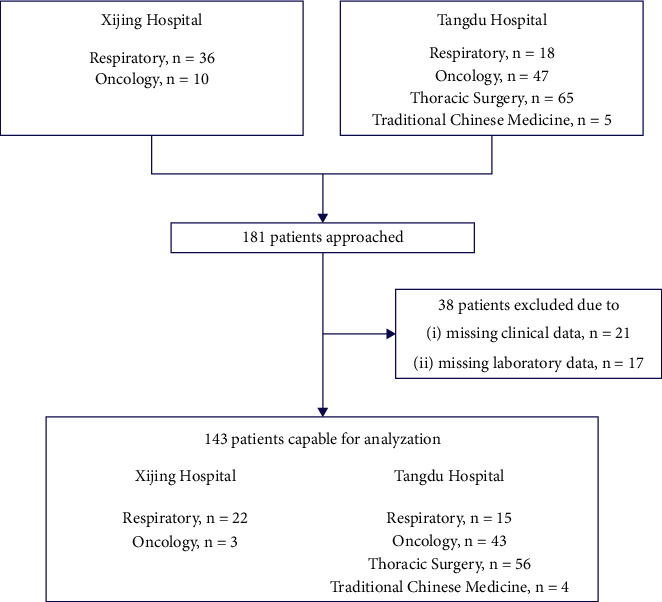
Study flowchart for ICIs cohort. Among the 181 NSCLC patients screened, 38 (21%) were excluded due to missing clinical or laboratory data.

We conducted a multicentre retrospective study of a cohort of patients with NSCLC receiving ICIs from 6 departments at 2 academic centers, the respiratory (*n* = 22) and oncology (*n* = 3) departments of Xijing Hospital and the respiratory (*n* = 15), oncology (*n* = 43), thoracic surgery (*n* = 56) and Traditional Chinese medicine (*n* = 4) departments of the Tangdu Hospital (Figure 1). The patient collection was based on the following inclusion criteria: (1) adult patients over 18 years old; (2) patients, who were pathologically diagnosed with NSCLC; (3) at least one radiological assessment per Response Evaluation Criteria in Solid Tumors (RECIST) v1.1 [16]; and (4) patients, who received ICIs. Patients, who matched any of the following criteria were excluded: (1) patients, who had ongoing noncancer related inflammation, immune disease, end-stage liver disease, or hematologic disease within 1 week before treatment; (2) patients with EGFR mutation or ALK and ROS1 gene fusion; (3) patients with other previous or concomitant cancers; and (4) patients with allergies or intolerance to ICIs or chemotherapy. A total of 143 patients from the Xijing Hospital (*n* = 25) and the Tangdu Hospital (*n* = 118) treated with ICIs between January 2018 and July 2019 were enrolled in the immunotherapy cohort and followed up until July 2021. A control cohort of 84 patients with NSCLC from the Xijing Hospital was exclusively treated with chemotherapy between June 2014 and April 2015.

### 2.2. Parameters and Assessments

Peripheral blood cell counts and ALB levels at baseline before ICI treatment were extracted from electronic medical records. Demographic, clinical, pathological, and molecular data were also collected. PD-L1 expression was analyzed on tumor cells by immunohistochemistry, according to the standard practice for each center. Expression of at least 1% was considered positive.

Radiological assessments were performed every 6 weeks as per RECIST v1.1 [16] as per the investigator's discretion in the immunotherapy cohort and the chemotherapy cohort. The objective remission rate (ORR) refers to the percentage of complete responses (CR) + partial responses (PR) patients out of the total number of patients, and the disease control rate (DCR) refers to the percentage of CR + PR + stable disease (SD) patients out of the total number of patients. OS was calculated from the date of initial immunotherapy administration until death (event) owing to any cause or the last follow-up (censored). PFS was calculated from the date of initial immunotherapy administration until disease progression or death (event) due to any cause.

### 2.3. Statistical Analysis

The dNLR was calculated as follows: dNLR = ANC/(WBC−ANC) [15]. The optimal cutoff value for the dNLR was greater than 3 and the ALB level was lower than 35 g/L based on previous largest published studies [15, 17]. The chi-square test and Fisher's exact test were used to analyze the distribution of clinical characteristics data. Significant parameters identified in univariate analysis (*P* < 0.05) were incorporated into multivariate Cox regression analysis to determine the independent factors associated with OS and PFS, and the hazard ratio (HR) was calculated. Variables associated with DCR were identified with logistic regression in the final multivariate model and were selected according to statistical significance in univariate analysis (*P* < 0.05), and the odds ratio (OR) was calculated. The *α* level was 5%. The results are presented as HR and OR and with 95% confidence interval (CI). Survival analyses were performed using the Kaplan–Meier diagram and compared by the log-rank method. All *P* < 0.05 were considered statistically significant. Data analysis was performed using SPSS software (version 22, IBM) and GraphPad Prism 8 software.

## 3. Results

### 3.1. Baseline Characteristics of the ICIs Cohort

The demographic and clinicopathological characteristics of the 143 patients with NSCLC receiving ICIs are given in Table 1. The patients ranged in age between 27 and 84 years old, with a median age of 63 years old. A total of 119 patients (83.2%) were male; 106 (74.1%) were smokers; 73 (51.0%) had adenocarcinoma, and 61 (42.7%) had squamous carcinoma. Among 32 (22.4%) patients with PD-L1 data, 24 (16.8%) had PD-L1 of at least 1% by immunohistochemical analysis, and 8 (5.6%) had negative results. Patients treated with ICIs, including sintilimab in 20 (14.0%) patients, nivolumab in 37 (25.9%) patients, and pembrolizumab in 86 (60.1%). A total of 46 (32.2%) patients were treated with ICIs monotherapy and 97 (67.8%) patients with ICIs combination therapy. A total of 46 (32.2%) patients were treated with ICIs as first-line, and 97 (67.8%) patients were treated with ICIs as a second or subsequent line.

### 3.2. dNLR and ALB

In the ICIs cohort (*n* = 143), the median follow-up was 13.3 months (95% CI, 12.7–13.9 months). The median PFS was 6.2 months (95% CI, 5.2–7.1 months), and the 1-year survival rates were 66.2% as the median OS was not reached. In disease response, CR was achieved in 2 patients (1.4%), PR was achieved in 60 patients (42.0%), SD was achieved in 55 patients (38.5%), progressed disease (PD) was achieved in 26 patients (18.2%), ORR was 43.4%, and DCR was 81.8%.

In the univariate analysis of the Cox regression model, ALB <35 g/L, dNLR >3 and metastatic sites number ≥2 were risk factors for PFS (HR 1.54, 95% CI 1.49–4.34; *P*=0.001; HR 1.92, 95% CI 1.17–3.15; *P*=0.010; HR 1.76, 95% CI 1.11–2.78; *P*=0.016), while ALB <35 g/L, dNLR >3, metastatic sites number ≥2 and squamous cell carcinoma were risk factors for OS (HR 4.48, 95% CI 2.12–9.47; *P* < 0.001; HR 2.16, 95% CI 1.02–4.54; *P*=0.044; HR 2.23, 95% CI 1.11–4.75; *P*=0.024; HR 2.70, 95% CI 1.32–5.52; *P*=0.006). In a multivariate analysis, the ALB <35 g/L and dNLR >3 were independent risk factors for PFS (HR 2.32, 95% CI 1.34–4.00; *P*=0.003; HR 1.71, 95% CI 1.03–2.85; *P*=0.037), the ALB <35 g/L, metastatic sites number ≥2 and squamous cell carcinoma were independent risk factors for OS (HR 3.90, 95% CI 1.77–8.64; *P*=0.001; HR 2.44, 95% CI 1.08–5.54; *P*=0.003; HR 4.22, 95% CI 1.97–9.04; *P* < 0.001) (Table 2). In a univariate analysis of the logistic regression model, ALB <35 g/L and ICIs line ≥ 2 were risk factors for DCR (OR 5.63, 95% CI 2.01–8.73; *P*=0.001; OR 4.10, 95% CI 1.36–9.30; *P*=0.018). In a multivariate analysis, ALB <35 g/L and ICIs line ≥ 2 were independent risk factors for DCR (OR 5.52, 95% CI 1.89–9.18; *P*=0.002; OR 5.99, 95% CI 1.29–9.71; *P*=0.022) (Table 3).

**Figure 2:**
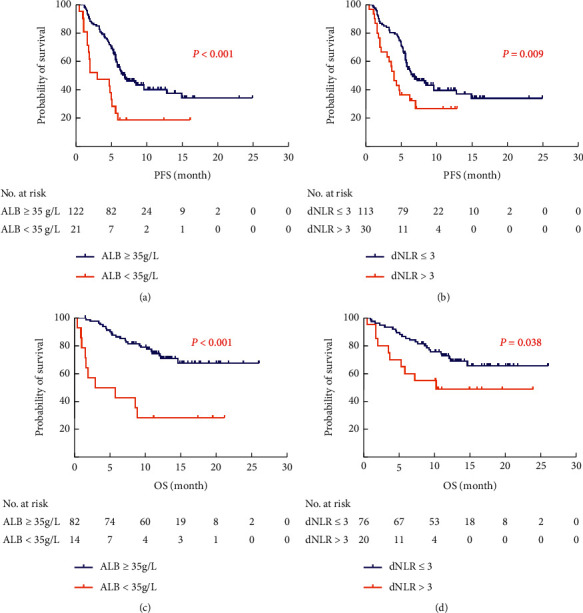
Kaplan–Meier curves of PFS and OS with regard to ALB and dNLR. (a) Kaplan–Meier curves of PFS with regard to ALB. (b) Kaplan–Meier curves of PFS with regard to dNLR. (c) Kaplan–Meier curves of OS with regard to ALB. (d) Kaplan–Meier curves of OS with regard to dNLR.

In the Kaplan–Meier survival analyses, the ALB <35 g/L and dNLR >3 were correlated with worse PFS (3.0 months vs. 6.9 months, *P* < 0.001; 4.0 months vs. 6.6 months, *P* = 0.009) and 1-year survival rates (28.6% vs. 72.8%, *P* < 0.001; 48.9% vs. 70.9%, *P*=0.038) compared with ALB ≥35 g/L and dNLR ≤3 (Figure 2).

### 3.3. Immune Checkpoint Inhibitor Prognostic Index (ICPI)

The ALB and dNLR were vital for the prognoses of NSCLC patients receiving ICIs. However, the predictive ability of individual indicators is relatively weak, a new prognostic indicator ICPI, based on the ALB <35 g/L and dNLR >3, had been constructed as a result. The ICPI was developed to characterize two groups, the low-risk ICPI (0 factor) and moderately high-risk ICPI (1 or 2 factors).

**Figure 3:**
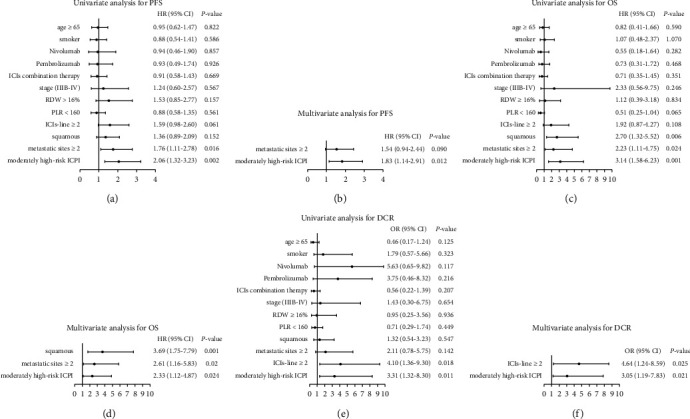
Univariate and multivariate analyses in the ICIs cohort: HR for PFS and OS, and OR for DCR (model 1: age, smoking status, metastatic sites number, ICIs line, histology, stage, RDW, PLR, and ICPI divided into 2 groups). (a) Univariate analysis for PFS. (b) Multivariate analysis for PFS. (c) Univariate analysis for OS. (d) Multivariate analysis for OS. (e) Univariate analysis for DCR. (f) Multivariate analysis for DCR.

**Figure 4:**
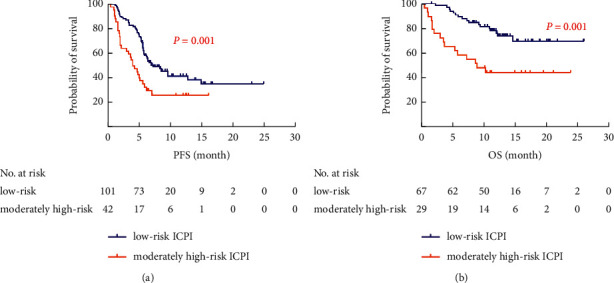
Kaplan–Meier curves of PFS and OS with regard to ICPI (divided into 2 groups). (a) Kaplan–Meier curves of PFS with regard to ICPI. (b) Kaplan–Meier curves of OS with regard to ICPI.

Among the 143 evaluable patients, 101 (71%) had low-risk ICPI, and 42 (29%) had moderately high-risk ICPI.  Table 4 provides baseline data including gender, age, pathological classification, KRAS mutation status, PD-L1 expression status, PS score, staging, ICIs line, and other data that showed no statistical significance in the distribution between the two groups (*P* > 0.05). In a multivariate analysis, the moderately high-risk ICPI was associated with significantly shorter PFS and OS (HR 1.83, 95% CI 1.14–2.91; *P*=0.012; HR 2.33, 95% CI 1.12–4.87; *P*=0.024, respectively), than the low-risk ICPI (Figure 3). The moderately high-risk ICPI and ICIs as second or subsequent line were also associated with progressive disease (non-DCR) (OR 3.05, 95% CI 1.19–7.83; *P*=0.021; OR 4.64, 95% CI 1.24–8.59; *P*=0.025, respectively) (Figure 3). The median PFS and OS for patients with moderately high-risk ICPI were shorter than that of low-risk ICPI (4.0 months *vs*. 7.2 months, *P*=0.001; 1-year survival rates: 44.3% *vs*. 76.1%, *P*=0.001) (Figure 4).

**Figure 5:**
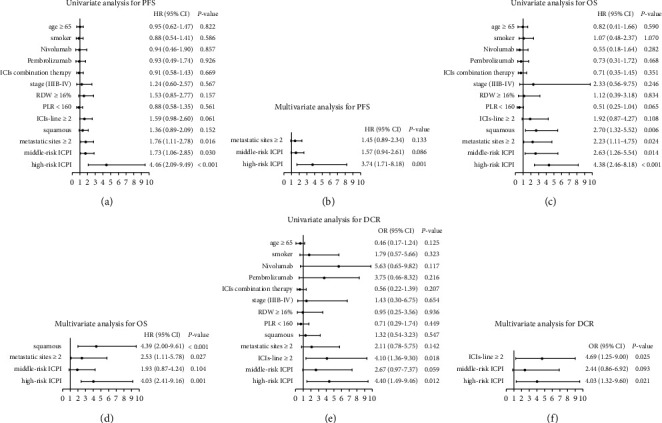
Univariate and multivariate analyses in the ICIs cohort: HR for PFS and OS, and OR for DCR (model 2: age, smoking status, metastatic sites number, ICIs line, histology, stage, RDW, PLR, and ICPI divided into 3 groups). (a) Univariate analysis for PFS. (b) Multivariate analysis for PFS. (c) Univariate analysis for OS. (d) Multivariate analysis for OS. (e) Univariate analysis for DCR. (f) Multivariate analysis for DCR.

**Figure 6:**
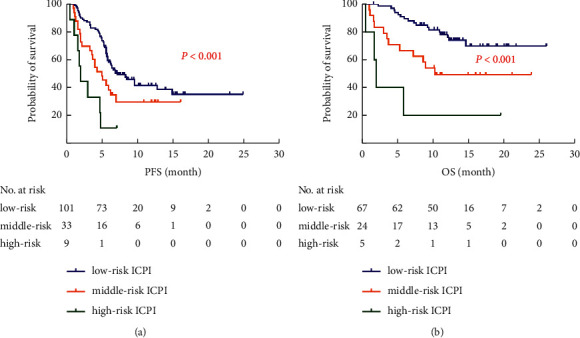
Kaplan–Meier curves of PFS and OS with regard to ICPI (divided into 3 groups). (a) Kaplan–Meier curves of PFS with regard to ICPI. (b) Kaplan–Meier curves of OS with regard to ICPI.

According to the ALB <35 g/L and dNLR >3, the ICPI was further divided into three groups, the low-risk ICPI (0 factor, *n* = 101) and middle-risk ICPI (1 factor, *n* = 33) and high-risk ICPI (2 factors, *n* = 9). In multivariate analysis, the high-risk ICPI was more significantly associated with worse PFS (HR 3.74, 95% CI 1.71–8.18; *P*=0.001), OS (HR 4.03, 95% CI 2.41–9.16; *P*=0.001), and DCR (OR 4.03, 95% CI 1.32–9.60; *P*=0.021), than the low-risk ICPI (Figure 5). The median PFS and OS for patients with the high-risk ICPI were shorter than the middle-risk and low-risk ICPI (2.0 months vs. 5.0 months vs. 7.2 months, *P* < 0.001; 1-year survival rates: 20.0% vs. 49.2% vs. 76.1%, *P* < 0.001) (Figure 6).

### 3.4. Chemotherapy Control Cohort

Whether ICPI was divided into two groups or three groups, the moderately high-risk ICPI or the high-risk ICPI was correlated with worse PFS, OS and DCR for NSCLC patients receiving ICIs. Therefore, this study further explored the predictive value of ICPI in NSCLC patients receiving chemotherapy. In the chemotherapy cohort, the 84 patients with lung cancer had a median follow-up of 8.7 months (95% CI 8.2–9.2 months). The median PFS and OS were 4.3 months (95% CI 2.6–6.0 months) and 11.1 months (95% CI 7.6–14.6 months). Baseline characteristics are given in Table 5.

**Figure 7:**
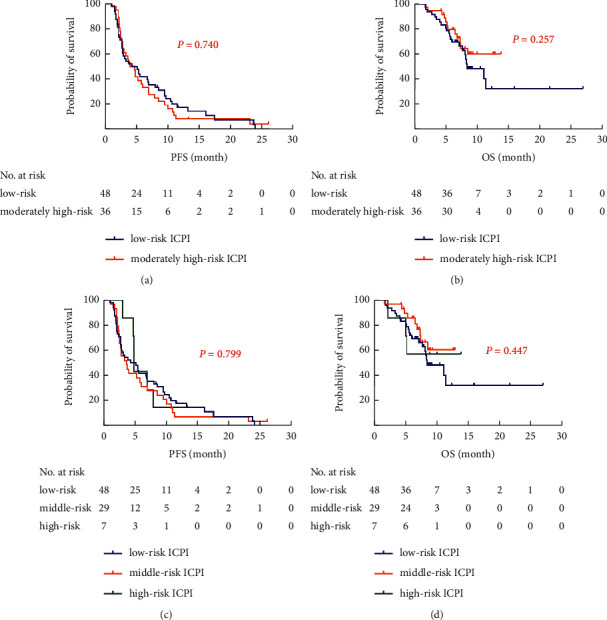
Kaplan–Meier curves of PFS and OS with regard to ICPI in NSCLC patients with chemotherapy. (a) Kaplan–Meier curves of PFS with regard to ICPI (divided into 2 groups). (b) Kaplan–Meier curves of OS with regard to ICPI (divided into 2 groups). (c) Kaplan–Meier curves of PFS with regard to ICPI (divided into 3 groups). (d) Kaplan–Meier curves of OS with regard to ICPI (divided into 3 groups).

When the ICPI was divided into two groups, 48 (57%) patients had a low-risk ICPI and 36 (43%) had a moderately high-risk ICPI. In contrast to the ICIs cohort, no significant differences in PFS and OS were observed among the moderately high-risk ICPI and low-risk ICPI in the chemotherapy cohort (4.0 months *vs*. 4.3 months, *P*=0.740; 1-year survival rates: 60.0% *vs.* 32.4%, *P*=0.257). The ICPI was further divided into 3 groups, the median PFS was 4.8 months *vs*. 3.6 months *vs*. 4.3 months (*P*=0.799), and 1-year survival rate was 57.1% *vs*. 60.7% *vs*. 32.4% (*P*=0.447) for the low-risk ICPI, middle-risk ICPI, and high-risk ICPI, respectively (Figure 7). In terms of DCR, whether ICPI was divided into two or three groups, the DCR was all 100%, so there was no significant difference between different ICPI groups.

## 4. Discussion

In our 143 patients treated with ICIs, the median PFS was 6.2 months (95% CI 5.2–7.1 months), which was similar to the PFS of the Impower 131 [18], Impower 130 [19], and KEYNOTE 407 [20]. The median OS did not reach, the reason might be as follows: first, the proportion of ICIs as first-line was high (32.2%); second, some patients received surgical treatment (35.0%) before ICIs treatment; Last, the period some patients assessed was not every 6 weeks as advised. Although the median OS did not reach in the present study, the 1-year survival rate was 66.2%, which was basically consistent with 61.3% in the Krefting study [21].

In the present study, multivariate analysis showed that the ALB <35 g/L was correlated with shorter PFS (3.0 months *vs*. 6.9 months, *P* < 0.001) and 1-year survival rates (28.6% *vs*. 72.8%, *P* < 0.001) compared with ALB ≥35 g/L in NSCLC receiving ICIs, which is consistent with previous findings that high ALB levels are associated with poor outcomes in various cancers, including melanoma, pancreatic cancer, lung cancer, gastric cancer, and breast cancer [22]. Kazandjian [17] et al. found that ALB <35 g/L was associated with poor OS and PFS in NSCLC receiving ICIs. This may be related to the following factors: first, for the host, the tumor is accompanied by tumor hypoxia and necrosis, and local tissue damage. In response to these changes, the body system releases proinflammatory cytokines and growth factors, and liver cells increase the production of acute phase proteins, such as CRP, and reduce ALB production [23]; second, liver synthesis of ALB is mainly affected by colloid osmotic pressure and inflammatory state but does not change in nutrient intake and malnutrition state [22, 24]. Therefore, hypoproteinaemia represents a proinflammatory state rather than a nutritional status in cancer patients [22]. A large number of pieces of evidence showed that hypoproteinaemia has also been found to be associated with a poor prognosis of NSCLC [15, 17]. In a multivariate analysis of the present study, the dNLR >3 was correlated with worse PFS (4.0 months *vs*. 6.6 months, *P*=0.009) and 1-year survival rates (48.9% *vs*. 70.9%, *P*=0.038) than dNLR ≤3, which is consistent with previous studies in patients with NSCLC treated with ICIs [15]. As an inflammatory response cell, neutrophil inhibits antitumor immune response by inhibiting the cytotoxic activity of immune cells, especially activated T cells [25]. The reduction of lymphocytes weakens the effect of ICIs and mainly releases the inhibitory signal of T cell function [25]. Therefore, researchers proposed the NLR, neutrophil-to-lymphocyte ratio. The prognostic value of NLR has been proven in various types of cancer [26–29]. Bagley [26] and Soyano [27] argued that high NLR was significantly associated with poor OS and PFS in NSCLC patients receiving ICIs. However, NLR only involves neutrophils and lymphocytes but does not involve monocytes (MON) and other granulocyte subsets. Therefore, researchers proposed the concept of dNLR. Mezquita [15] found that baseline dNLR >3 was associated with poor PFS and OS in patients with advanced NSCLC receiving ICIs (HR 1.83, 95% CI 1.12–2.98; *P*=0.015; HR 2.22, 95% CI 1.23–4.01; *P*=0.008). However, other studies showed no significant statistical difference in the correlation between high dNLR and PFS and OS (1.0 months *vs*. 4.0 months, *P*=0.924; 2.0 months *vs*. 6.0 months, *P*=0.789) [30], which may be related to the duality of neutrophil [25, 31, 32].

In the last 15 years, there has been a movement towards the use of combined prognostic scores [33–35]. Since ALB <35 g/L and dNLR >3 were closely associated with unfavorable prognosis in NSCLC patients treated with ICIs, we constructed a new prognostic index, ICPI, based on the two risk factors. The results showed that the ICPI was correlated with worse PFS, OS and DCR for NSCLC patients receiving ICIs. The moderately high-risk ICPI had a significantly increased risk of progression, death, and non-DCR (*P* < 0.05), and had worse PFS and 1-year survival rates (4.0 months *vs.* 7.2 months, *P*=0.001; 44.3% *vs.* 76.1%, *P*=0.001) compared with low-risk ICPI. Similarly, in further analysis, the ICPI was divided into three groups, and the results demonstrated that the high-risk ICPI was correlated with worse PFS and 1-year survival rates compared with middle-risk ICPI and low-risk ICPI (2.0 months *vs.* 5.0 months *vs.* 7.2 months, *P* < 0.001; 20.0% *vs.* 49.2% *vs.* 76.1%, *P* < 0.0011). However, there were only 9 low-risk ICPI patients (6%), which may impact the results, and requires validation in external populations. On the other hand, the ICPI was not associated with outcomes in patients treated with chemotherapy only. Previous studies have also combined different indicators, such as the number of metastatic sites, gastrointestinal tumors, PS score, age, platelet, neutrophil, absolute lymphocyte counts, LDH, ALB and NLR, and so on to form a new prognostic scoring system [36, 37]. For example, Mezquita [15] proposed LIPI, which is defined as the combination of dNLR >3 and LDH > upper limit of normal, and divided LIPI into three groups, good LIPI (0 factor), intermediate LIPI (1 factor), and poor LIPI (2 factors). The results showed that the good LIPI had longer PFS and OS than the intermediate LIPI and poor LIPI (6.3 months *vs*. 3.7 months *vs*. 2.0 months; 34 months *vs*. 10 months *vs*. 3 months, both *P* < 0.001), and there was no significant correlation between this index and the prognosis of chemotherapy, which was consistent with the results of the present study.

In the present study, a total of 143 NSCLC patients received ICIs treatment, PD-L1 expression was tested in 32 patients (22.4%), among which 24 patients (16.8%) were positive (PD-L1 ≥ 1%) and 8 patients (5.6%) were negative, and therefore 111 patients (77.6%) had unknown expression status. Mezquita [15] also had an unknown PD-L1 expression status (72%). This may not affect the results of this study, because PD-L1 testing was not mandatory at that time, and most patients received second or subsequent line treatment. Moreover, KEYNOTE189 [38] and CheckMate 017 [7] both describe that regardless of PD-L1 expression level or even negative, NSCLC patients showed clinical benefits from ICIs treatment.

However, there are some limitations in this study. First, the present study was a retrospective evaluation with potential biases due to missing trials or missing laboratory values, such as the LDH level and ECOG PS. Second, the identified cutoff values for the dNLR and ALB need to be validated in external populations. Third, the information of some patients including concurrent conditions and medications is missing. Comorbidities (such as infections or inflammation) and the use of steroids that may cause changes in peripheral blood cell counts are lacking. Future modeling could incorporate other known prognostic markers such as the performance status, other baseline factors, tumor genomic, transcriptomic, proteomic, and metabolomic markers.

## 5. Conclusions

The ALB <35 g/L and dNLR >3 were correlated with worse PFS and OS for NSCLC patients receiving ICIs. The ICPI was correlated with an unfavorable prognosis for NSCLC patients receiving ICIs, but not for patients with chemotherapy, suggesting that baseline ICPI might be useful for identifying patients, who are unlikely to benefit from treatment with ICIs and avoiding unnecessary immunotoxicity and financial toxicity.

## Figures and Tables

**Table 1:** The baseline characteristics of the ICIs cohort.

Patients (*n* = 143)	
Sex	
Male	119 (83.2)

Age (year)	
≥65	57 (39.9)

Smoking status	
Nonsmoker	37 (25.9)
Smoker	106 (74.1)

Histology	
Adenocarcinoma	73 (51.0)
Squamous	61 (42.7)
NSCLC-others	9 (6.3)

KRAS alteration status	
KRAS wild-type	65 (45.5)
KRAS mutant	6 (4.2)
NA	72 (50.3)

PD-L1 status	
Negative	8 (5.6)
Positive	24 (16.8)
NA	111 (77.6)

PS (ECOG)	
0-1	141 (98.6)
≥2	2 (1.4)

Stage	
I-II	7 (4.9)
IIIA	9 (6.3)
IIIB–IV	127 (88.8)

Metastatic sites number	
<2	57 (39.9)
≥2	86 (60.1)

Metastatic sites	
Live	18 (12.6)
Bone	34 (23.8)
Brain	19 (13.3)
WBC (**×**10^9^/L)	6.62 (5.51–8.87)
ANC (**×**10^9^/L)	4.36 (3.09–6.01)
ALC (**×**10^9^/L)	1.46 (1.02–1.82)
MON (**×**10^9^/L)	0.59 (0.42–0.81)
RDW (%)	13.8 (13.1–15.0)
PLT (**×**10^9^/L)	230 (174–296)
ALB (g/L)	40.89 ± 4.85
PLR	155.62 (117.56–227.74)
dNLR	2.08 (1.45–2.74)

ICIs drug	
Sintilimab	20 (14.0)
Nivolumab	37 (25.9)
Pembrolizumab	86 (60.1)

ICIs treatment modality	
ICI monotherapy	46 (32.2)
ICI + chemotherapy	87 (60.8)
ICI + antiangiogenic	10 (7)

ICIs line	
1	46 (32.2)
≥2	97 (67.8)

Previous treatments before ICIs	
Chemotherapy	89 (62.2)
Radiotherapy	23 (16.1)
EGFR-TKI	12 (8.4)
Antiangiogenic	25 (17.5)
Surgery	13 (9.1)

Disease response	
CR	2 (1.4)
PR	60 (42.0)
SD	55 (38.5)
PD	26 (18.2)

Response rates	
ORR (%)	43.4
DCR (%)	81.8

NA, not assessable; MON, monocyte.

**Table 2:** The univariate and multivariate analyses in the ICIs cohort: HR for PFS and OS.

Variable	PFS				OS			
	Univariate		Multivariate		Univariate		Multivariate	
	HR (95% CI)	*P* value	HR (95% CI)	*P* value	HR (95% CI)	*P* value	HR (95% CI)	*P* value
Age (year)								
<65	1				1			
≥65	0.95 (0.62–1.47)	0.822			0.82 (0.41–1.66)	0.590		

Smoking status								
Nonsmoker	1				1			
Smoker	0.88 (0.54–1.41)	0.586			1.07 (0.48–2.37)	1.070		

Metastatic sites number								
<2	1		1		1		1	
≥2	1.76 (1.11–2.78)	0.016	1.43 (0.88–2.33)	0.144	2.23 (1.11–4.75)	0.024	2.44 (1.08–5.54)	0.033

ICIs-drug								
Sintilimab	1				1			
Nivolumab	0.94 (0.46–1.90)	0.857			0.55 (0.18–1.64)	0.282		
Pembrolizumab	0.93 (0.49–1.74)	0.926			0.73(0.31–1.72)	0.468		

ICIs treatment modality								
Monotherapy	1				1			
Combination therapy	0.91 (0.58–1.43)	0.669			0.71 (0.35–1.45)	0.351		

ICIs line								
1	1				1			
≥2	1.59 (0.98–2.60)	0.061			1.92 (0.87–4.27)	0.108		

Histology								
Nonsquamous	1				1		1	
Squamous	1.36 (0.89–2.09)	0.152			2.70 (1.32–5.52)	0.006	4.22 (1.97–9.04)	<0.001

Stage								
I–IIIA	1				1			
IIIB–IV	1.24 (0.60–2.57)	0.567			2.33 (0.56–9.75)	0.246		

RDW (%)								
<16	1				1			
≥16	1.53 (0.85–2.77)	0.157			1.12 (0.39–3.18)	0.834		

LDH (IU/L)								
<250	1				1			
≥250	2.32 (1.22–4.40)	0.010			2.23 (0.80–6.21)	0.124		

ALB (g/L)								
≥35	1		1		1		1	
<35	1.54 (1.49–4.34)	0.001	2.32 (1.34–4.00)	0.003	4.48 (2.12–9.47)	<0.001	3.90 (1.77–8.64)	0.001

dNLR								
≤3	1		1		1		1	
>3	1.92 (1.17–3.15)	0.010	1.71 (1.03–2.85)	0.037	2.16 (1.02–4.54)	0.044	1.70 (0.78–3.70)	0.18

PLR								
≥160	1				1			
<160	0.88 (0.58–1.35)	0.561			0.51 (0.25–1.04)	0.065		

RDW, red blood cell distribution width; PLR, platelet-to-lymphocyte ratio.

**Table 3:** The univariate and multivariate analyses in the ICIs cohort: OR for DCR.

Variable	Univariate		Multivariate	
	OR (95% CI)	*P* value	OR (95% CI)	*P* value
Age (year)				
<65	1			
≥65	0.46 (0.17–1.24)	0.125		

Smoking status				
Nonsmoker	1			
Smoker	1.79 (0.57–5.66)	0.323		

Metastatic sites number				
<2	1			
≥2	2.11 (0.78–5.75)	0.142		

ICIs drug				
Sintilimab	1			
Nivolumab	5.63 (0.65–9.82)	0.117		
Pembrolizumab	3.75 (0.46–8.32)	0.216		

ICIs treatment modality				
Monotherapy	1			
Combination therapy	0.56 (0.22–1.39)	0.207		

ICIs line				
1	1		1	
≥2	4.10 (1.36–9.30)	0.018	5.99 (1.29–9.71)	0.022

Histology				
Nonsquamous	1			
Squamous	1.32 (0.54–3.23)	0.547		

Stage				
I–IIIA	1			
IIIB–IV	1.43 (0.30–6.75)	0.654		

RDW (%)				
<16	1			
≥16	0.95 (0.25–3.56)	0.936		

ALB (g/L)				
≥35	1		1	
<35	5.63 (2.01–8.73)	0.001	5.52 (1.89–9.18)	0.002

dNLR				
≤3	1			
>3	1.89 (1.69–5.15)	0.213		

PLR				
≥160	1			
<160	0.71 (0.29–1.74)	0.449		

**Table 4:** The baseline characteristics according to the ICPI group in the ICIs cohort.

	Low-risk ICPI	Moderately high-risk ICPI	*P* value
	*n* = 101	*n* = 42	
Sex			0.314
Male	19 (18.8)	5 (11.9)	

Age (year)			0.923
≥65	40 (39.6)	17 (40.5)	

Smoking status			0.031
Nonsmoker	21 (20.8)	16 (38.1)	
Smoker	80 (79.2)	26 (61.9)	

Histology			0.960
Adenocarcinoma	52 (51.5)	21 (50.0)	
Squamous	43 (42.6)	18 (42.9)	
NSCLC-others	6 (5.9)	3 (7.1)	

KRAS alteration status			0.496
KRAS wild-type	43 (42.6)	22 (52.4)	
KRAS-mutant	5 (5.0)	1 (2.4)	
NA	53 (52.5)	19 (45.2)	

PD-L1 status			0.622
Negative	6 (5.9)	2 (4.8)	
Positive	15 (14.9)	9 (21.4)	
NA	80 (79.2)	31 (73.8)	

PS (ECOG)			0.085
0-1	101 (100)	40 (95.2)	
≥2	0 (0)	2 (4.8)	

Stage			0.090
I-II	7 (6.9)	0 (0)	
IIIA	8 (7.9)	1 (2.4)	
IIIB–IV	86 (85.1)	41 (97.8)	

Metastatic sites number			<0.001
<2	50 (49.5)	7 (16.7)	
≥2	51 (50.5)	35 (83.3)	

Metastatic sites			
Live	9 (8.9)	9 (21.4)	0.04
Bone	16 (15.8)	18 (42.9)	0.001
Brain	14 (13.9)	5 (11.9)	0.754
WBC (**×**10^9^/L)	6.44 (5.22–7.75)	8.11 (6.09–10.13)	<0.001
ANC (**×**10^9^/L)	4.04 (2.81–5.10)	6.02 (4.69–8.00)	<0.001
ALC (**×**10^9^/L)	1.60 (1.13–1.95)	1.01 (0.81–1.54)	<0.001
MON (**×**10^9^/L)	0.54 (0.39–.071)	0.61 (0.41–0.75)	0.879
RDW (%)	13.4 (13.0–14.6)	14.6 (13.6–14.9)	0.150
PLT (**×**10^9^/L)	231 (163–289)	212 (180–309)	0.929
ALB (g/L)	42.93 ± 3.61	36.49 ± 4.26	<0.001
PLR	139.0 (110.1–198.4)	218.5 (144.2–284.5)	<0.001
dNLR	1.93 (1.16–2.28)	3.62 (2.51–4.31)	<0.001

ICIs drug			0.028
Sintilimab	18 (90)	2 (10)	
Nivolumab	21 (56.8)	16 (43.2)	
Pembrolizumab	62 (72.1)	24 (27.9)	

ICIs treatment modality			0.011
ICI monotherapy	26 (56.5)	20 (43.5)	
ICI + chemotherapy	75 (77.3)	22 (22.7)	
ICI + antiangiogenic			

ICIs line			0.168
1	36 (35.6)	10 (23.8)	
≥2	65 (64.4)	32 (76.2)	

Previous treatments			
Chemotherapy	61 (60.4)	28 (66.7)	0.481
Radiotherapy	11 (10.9)	12 (28.6)	0.009
EGFR-TKI	8 (7.9)	4 (9.5)	0.753
Antiangiogenic	16 (15.8)	9 (21.4)	0.423
Surgery	9 (8.9)	4 (9.5)	0.156

Disease response			0.103
CR	2 (2.0)	0 (0)	
PR	44 (43.6)	16 (38.1)	
SD	42 (41.6)	13 (31.0)	
PD	13 (12.9)	13 (21.0)	

Response rates			
ORR (%)	45.5	38.1	0.714
DCR (%)	87.1	69	0.031

**Table 5:** The baseline characteristics according to ICPI groups in the chemotherapy cohort.

	All patients	Low-risk ICPI	Moderately high-risk ICPI	*P* value
	*n* = 84	*n* = 48	*n* = 36	
Sex				0.547
Male	65 (77.4)	36 (75.5)	29 (80.6)	

Age (year)				0.842
≥65	29 (34.5)	17 (35)	12 (33)	

Smoking status				0.500
Nonsmoker	32 (38.1)	20 (41.7)	12 (33.3)	
Smoker	52 (61.9)	28 (58.3)	24 (66.7)	

Histology				0.236
Adenocarcinoma	47 (56.0)	25 (52.1)	22 (61.1)	
Squamous	33 (39.3)	19 (39.6)	14 (38.9)	
NSCLC-others	4 (4.8)	4 (4.8)	0 (0)	

KRAS alteration status				0.588
KRAS wild-type	53 (63.1)	28 (58.3)	25 (69.4)	
KRAS-mutant	4 (4.8)	3 (6.3)	1 (2.8)	
NA	27 (32.1)	17 (35.4)	10 (27.8)	

PD-L1 status				
Negative				
Positive				
NA				

PS (ECOG)				
0-1	84 (100)	48 (100)	36 (100)	
≥2	0	0	0	

Stage				0.037
I-II	1 (1.2)	0 (0)	1 (2.8)	
IIIA	5 (6.0)	5 (10.4)	0 (0)	
IIIB–IV	78 (92.9)	43 (89.6)	35 (97)	

Metastatic sites number				0.042
<2	32 (38.1)	23 (47.9)	9 (25.0)	
≥ 2	52 (61.9)	25 (52.1)	27 (75)	

Metastatic sites				
Live	2 (2.4)	1 (2.1)	1 (2.8)	0.836
Bone	17 (20.2)	10 (20.8)	7 (19.4)	0.875
Brain	12 (14.3)	5 (10.4)	7 (19.4)	0.242
WBC (**×**10^9^/L)	7.41 ± 2.19	7.05 ± 1.96	7.87 ± 2.42	0.091
ANC (**×**10^9^/L)	5.19 ± 1.91	4.62 ± 1.57	5.95 ± 2.07	0.001
ALC (**×**10^9^/L)	1.44 ± 0.48	1.68 ± 0.42	1.13 ± 0.36	<0.001
MON (**×**10^9^/L)	0.48 (0.37–0.61)	0.47 (0.37–0.57)	0.50 (0.40–0.67)	0.183
RDW (%)	13.2 (12.7–13.8)	13.3 (12.7–14.0)	13.1 (12.6–13.78)	0.861
PLT (**×**10^9^/L)	235 (183–297)	230 (173–276)	259 (190–361)	0.054
ALB (g/L)	38.38 ± 4.87	40.71 ± 3.62	35.28 ± 4.62	<0.001
PLR	171.6 (119.1–231.4)	145.4 (108.4–180.0)	237 (183.1–338.9)	<0.001
dNLR	2.33 (1.77–3.06)	1.95 (1.56–2.35)	3.13 (2.39–3.97)	<0.001

Disease response				0.137
CR	0	0	0	
PR	70 (83.3)	37 (77.1)	33 (91.7)	
SD	14 (16.7)	11 (22.9)	3 (8.3)	
PD	0	0	0	
NA				

Response rates				0.139
ORR (%)	83.8	77.1	91.7	
DCR (%)	100	100	100	

## Data Availability

The datasets generated during and/or analyzed during the current study are available from the corresponding author upon request.
